# Association between Leukocyte Counts and Physical Fitness in Male Military Members: The CHIEF Study

**DOI:** 10.1038/s41598-020-63147-9

**Published:** 2020-04-08

**Authors:** Pei-Shou Chung, Kun-Zhe Tsai, Yen-Po Lin, Yu-Kai Lin, Gen-Min Lin

**Affiliations:** 1 Department of Medicine, Hualien Armed Forces General Hospital, Hualien, Taiwan; 2 Department of Critical Care Medicine, Taipei Tzu-Chi Hospital, New Taipei, Taiwan; 3 Departments of Neurology and Internal Medicine, Tri-Service General Hospital, National Defense Medical Center, Taipei, Taiwan; 4 Internal Medicine, Tri-Service General Hospital, National Defense Medical Center, Taipei, Taiwan; 5 Department of Preventive Medicine, Northwestern University Feinberg School of Medicine, Chicago, IL 60611 USA

**Keywords:** Risk factors, Chronic inflammation, Chronic inflammation, Risk factors

## Abstract

Low-grade inflammation, which is related to obesity and toxic substance use in young adults, may be associated with poor physical fitness. We investigated the association between total leukocyte count and physical fitness in a military cohort of 3,453 healthy young Taiwanese males aged 20–50 years in a cross-sectional study in 2014. Low-grade inflammation was defined according to equally sized quartiles of total leukocyte counts within the suggested normal limits (4.00–9.99 × 10^3^/mm^3^). Aerobic fitness was assessed by the time for a 3-kilometer run test, and anaerobic fitness was evaluated by the numbers of sit-ups and push-ups performed in 2 minutes. Automatic monitoring systems were used to verify the scores for all procedures. Multiple linear regression was utilized to identify the associations among variables. When compared with the lowest counts (4.00–5.49 × 10^3^/mm^3^), the second highest (6.50–7.49 × 10^3^/mm^3^) and highest normal leukocyte counts (7.50–9.99 × 10^3^/mm^3^) were correlated with longer times for a 3-kilometer run (β and 95% confidence intervals =4.93 (1.61, 8.25) and 4.65 (2.20, 7.10), respectively) and fewer numbers of push-ups performed in 2 minutes (β = −0.59 (−1.15, −0.03) and −0.56 (−0.96, −0.17), respectively), after adjustments for age, service specialty, waist circumference, body mass index, alcohol consumption, tobacco smoking, and physical activity. However, the association with 2-minute sit-ups was null. Our study suggested an inverse association between total leukocyte count and not only aerobic fitness but also parts of anaerobic fitness in young males. The temporal association needs confirmation in longitudinal studies.

## Introduction

Low-grade inflammation usually refers to a mild upregulation of cytokine and leukocyte levels in the blood^1,2^, and it is related to a more pronounced activation state of immune cells^3^. In the absence of a septic process and chronic diseases, proinflammatory cytokines such as tumor necrosis factor (TNF)-α and interleukin (IL)−1β and IL-6 can be generated by adipocytes^4,5^ and stimulate hepatocytes to produce C-reactive protein^6^, which further induces bone marrow to increase circulating blood leukocyte (white blood cells) concentrations^7^. Metabolic abnormalities, including overweight, obesity, hyperuricemia and atherosclerosis, have been associated with low-grade inflammation in the body^7^. In addition, exposure to toxic substances such as tobacco smoke and alcohol beverages can activate innate immunity^8^, leading to systemic inflammation. Furthermore, mental stress, such as anxiety and posttraumatic stress disorder, could increase high-sensitivity C-reactive protein and decrease adiponectin concentrations, where adiponectin is known as a cardio-protective marker^9,10^.

Several previous population studies including diabetic patients^11^, those with metabolic syndrome^12^, or apparently healthy middle- and old-aged individuals^14–16^ have revealed that the degree of low-grade inflammation evaluated by total leucocyte counts and C-reactive protein concentrations was dose-dependently and directly associated with body fatness but inversely associated with cardiorespiratory fitness, as assessed by the maximum oxygen consumption during a treadmill exercise test. However, studies on the association between low-grade inflammation and muscular strength (anaerobic fitness) are lacking, and few studies have examined the association with cardiorespiratory fitness in young adults.

To our knowledge, systemic low-grade inflammation in young adults is mainly associated with great body adiposity and habitual toxic substance use, and it is not easily evaluated in large population studies. Since physical fitness is highly related to the performance of exercises, we aimed to investigate the association of low-grade inflammation with aerobic and anaerobic fitness in a large military cohort of healthy young male adults who participated in annual exercise tests in eastern Taiwan.

## Methods

### Study population

The study subjects were obtained from a military population of 6,133 young adults, aged 18–50 years, from the cardiorespiratory fitness and hospitalization events in armed forces (CHIEF) study performed in Taiwan during 2014^17^. All participants carried out a health screening involving a physical examination, chest roentgenography, blood and urine tests in the Hualien Armed Forces General Hospital, and a self-reported questionnaire regarding the habits of cigarette smoking, alcohol intake, and betel nut chewing status (current vs. former or never) and total physical activity per week over the past 6 months. Before the laboratory studies, each participant’s temporal temperature and general condition were checked, and each patient was interviewed by a physician. If there were any infection symptoms or signs along with a body temperature over >37 °C, the participant’s next visit was scheduled after full recovery from the sickness was achieved. The inclusion criteria were the participants completing at least one of the three exercise tests, including sit-ups for 2 minutes, push-ups for 2 minutes and a 3000-meter run, for military awards or rank promotions at the Military Physical Testing Center in Hualien after the health screening in 2014. As a result, 4,080 participants were included, and the response rate was approximately 66.5%. The detailed design of the CHIEF study has been reported previously^17–24^. The exclusion criteria were females with an active menstrual cycle, which could influence all blood cell counts (n = 411), and those with total leukocyte counts <4,000 × 10^3^/mm^3^ (n = 36) or >10,000 × 10^3^/mm^3^ (n = 50) or with evidence for volume depletions such as recent blood loss, diarrhea and plasma blood urea nitrogen >20 mg/dL (n = 130). These criteria yielded a final sample of 3,453 males for analysis.

### Laboratory assessment

For each participant, body height and weight were assessed in a standing position. Body mass index was defined as the ratio of body weight (kilogram) to the square of height (meter^2^). Measurement of waist circumference was taken at the midpoint between the highest point of the iliac crests and the lowest point of palpable ribs. Blood pressure (BP) was measured after resting for at least 25 minutes and taken once from the right arm in a sitting position with an automated FT-201 BP monitor (Parama-Tech Co Ltd, Fukuoka, Japan). The metabolic panel of blood biochemical tests for serum triglycerides, fasting plasma glucose, low-density lipoprotein cholesterol, high-density lipoprotein cholesterol, and total cholesterol were measured on an AU640 autoanalyzer (Olympus, Kobe, Japan). Routine blood tests, including total white blood cell (leukocyte) counts, red blood cell counts, platelet counts and hemoglobin levels, were measured using an automated hematology analyzer (Sysmex XT- 2000-I, Sysmex America, IL, USA). All blood samples of the participants were collected after an overnight 12-hour fast at the same blood drawing station.

### Physical fitness assessment

The time for completing a 3000-meter run test, which is a strong indicator of the velocity at lactate (anaerobic) threshold, is used to evaluate the superiority of aerobic fitness^25^. The participants did not carry any objects during the test. All runs began at 4:00 PM and were performed on a flat rectangular playground only if there was no heavy rain and the risk coefficient of heat stroke (i.e., the outdoor temperature (°C) × the outdoor relative humidity (%) × 0.1) was lower than 40. In addition, the number of push-ups performed in 2 minutes and the number of sit-ups performed in 2 minutes were separately investigated for the superiority of anaerobic fitness. These two procedures were scored in a standard manner by computer infrared sensing systems. As the exercise tests are linked to rank promotion and awards, the best performance is considered the fitness of each participant. All testing courses were monitored by the observing officers and video recorded. This study was reviewed and approved by the Institutional Review Board of Mennonite Christian Hospital (No. 16-05-008) in Hualien, Taiwan, and written informed consent was obtained from all participants. All procedures were performed in accordance with the relevant guidelines and regulations.

### Statistical analysis

Systemic low-grade inflammation was graded by equally sized quartiles of total leukocyte counts within the normal limits as follows: the lowest leukocyte counts (4,000–5,499/mm^3^, N = 768), the second lowest leukocyte counts (5,500–6,499/mm^3^, N = 1,015), the second highest leukocyte counts (6,500–7,499/mm^3^, N = 842) and the highest leukocyte counts (7,499–9,999 × 10^3^/mm^3^, N = 828). For the baseline characteristics of each group, continuous data were expressed as the mean ± standard deviation (SD) and compared by two-tailed t test. When the normality test (Kolmogorov-Smirnov) indicated non-normality, the Wilcoxon signed rank test was used. Categorical data were expressed as numbers (%) and compared by χ^2^ test or Fisher’s exact test.

The differences in each exercise performance among those with the lowest, second lowest, second highest and highest leukocyte counts were estimated by using analysis of covariance (ANCOVA), and the results are presented as the mean ± standard error (SE). Multiple stepwise linear regressions were used to determine the correlation of the second lowest, second highest, and highest leukocyte counts with each exercise performance with the lowest leukocyte counts as the reference. In addition, given that the large sample size of the study could provide sufficient power for subgroup analysis, multiple linear regressions for total leukocyte count and each exercise performance were performed for those aged ≥30 years and those aged <30 years.

In model 1, age and service specialty were used to adjust the model. In model 2, body mass index was also used. In model 3, the covariates in model 2 and waist circumference were used to adjust the model. In model 4, smoking, alcohol intake status, and physical activity were also used. These potential confounders were chosen for the models according to prior published associations between low-grade inflammation and fitness^26,27^. A 2-tailed value of *p* < 0.05 was considered significant. SAS statistical software (SAS System for Windows, version 9.4; SAS Institute, Cary, NC, United States) was used for all statistical analyses.

## Results

### Baseline group characteristics

The baseline characteristics of each group are shown in Table 1. Within the normal limits of total leukocyte counts, males with higher total leukocyte counts were found to have greater body mass indexes, waist circumferences and atherogenic lipid profiles, and they had an increased prevalence of tobacco smoking and alcohol beverage intake. Notably, the mean ages and physical activity of the participants in the four groups were similar.

**Table 1:** Baseline Characteristics of Study Participants Stratified by Leukocyte Counts (n = 3,453).

Characteristics	WBC counts 0^th^–25^th^ % (n = 768)	WBC counts 25^th^–50^th^ % (n = 1,015)	WBC counts 50^th^–75^th^ % (n = 842)	WBC counts 75^th^–100^th^ % (n = 828)	*p*-value
Age (years)	29.6 ± 5.9	29.1 ± 5.9	29.2 ± 5.9	29.3 ± 5.8	0.34
**Specialty, %**					
Army	409 [53.3]	532 [52.4]	409 [48.6]	397 [47.9]	0.11
Navy	115 [20.2]	217 [21.4]	173 [20.5]	194 [23.4]	
Air force	204 [26.5]	266 [26.2]	260 [30.9]	237 [28.6]	
Body mass index (kg/m^2^)	24.3 ± 2.9	24.6 ± 3.0	25.0 ± 3.1	25.4 ± 3.2	<0.01
Waist circumference (cm)	81.8 ± 7.6	82.8 ± 7.8	83.7 ± 7.8	85.1 ± 8.1	<0.01
Systolic blood pressure (mmHg)	116.0 ± 12.9	118.1 ± 12.9	118.8 ± 13.1	120.4 ± 13.3	<0.01
Diastolic blood pressure (mmHg)	68.8 ± 9.9	70.5 ± 9.9	70.8 ± 10.2	71.9 ± 10.3	<0.01
**Blood test**					
Total cholesterol (mg/dL)	171.4 ± 32.2	171.2 ± 33.5	176.6 ± 35.0	178.0 ± 34.3	<0.01
Serum triglyceride (mg/dL)	91.6 ± 66.8	107.3 ± 113.5	123.8 ± 104.8	131.2 ± 3.2	<0.01
Fasting glucose (mg/dL)	92.7 ± 9.8	93.7 ± 14.1	93.7 ± 12.1	94.3 ± 16.1	0.10
HDL-C (mg/dL)	49.8 ± 10.3	48.2 ± 9.4	47.3 ± 9.6	46.5 ± 9.4	<0.01
LDL-C (mg/dL)	103.1 ± 28.0	103.6 ± 29.3	107.7 ± 30.1	109.5 ± 30.4	<0.01
WBC counts (10^3^/mm^3^)	4.9 ± 0.4	6.0 ± 0.3	7.0 ± 0.3	8.4 ± 0.7	<0.01
(WBC Range: Min-Max)	4.00–5.49	5.50–6.49	6.50–7.49	7.50–9.99	
Hemoglobin, (g/dL)	15.0 ± 0.9	15.1 ± 1.0	15.2 ± 1.0	15.4 ± 1.0	<0.01
**Alcohol intake status, %**					
Former or never	466 [60.7]	596 [58.7]	454 [53.9]	424 [51.2]	<0.01
Current	302 [39.3]	419 [41.3]	388 [46.1]	404 [48.8]	
**Tobacco smoking status, %**					
Never smoker	535 [70.8]	692 [69.1]	485 [58.4]	418 [51.4]	<0.01
Current smoker	221 [29.2]	310 [30.9]	346 [41.6]	395 [48.6]	
**Physical activity, %**					
Never or occasionally	151 [19.7]	204 [20.1]	192 [22.8]	174 [21.0]	0.75
1–2 times/week	287 [37.4]	387 [38.1]	314 [37.3]	307 [37.1]	
≥ 3 times/week	330 [43.0]	424 [41.8]	336 [39.9]	347 [41.9]	

Continuous variables are expressed as mean ± SD (standard deviation), and categorical variables as n [%].

Abbreviations: HDL-C, high-density lipoprotein cholesterol; LDL-C, low-density lipoprotein cholesterol, WBC: white blood cell.

### Group means comparisons

Table 2 reveals the ANCOVA results for testing the differences in exercise performance among groups. There were differences in the numbers of push-ups and sit-ups in 2 minutes and the time for a 3000-meter run test among the four groups in model 1 to model 3 (all overall p-values ≤0.01), indicating a trend towards low physical fitness and high leukocyte counts within normal limits hat is independent of age and body adiposity. Compared with the lowest normal leukocyte counts, the second highest and highest normal leukocyte counts had significantly lower numbers of push-ups in 2 minutes and longer times for a 3000-meter run in model 4. There were no differences among the four groups in the number of sit-ups in 2 minutes in model 4.

**Table 2:** Differences in Each Exercise Performance between Different Leukocyte Count Groups.

	WBC counts 0^th^–25^th^			WBC counts 25^th^–50^th^			WBC counts 50^th^–75^th^			WBC counts 75^th^–100^th^		
	n	Mean (SE)	*p*-value	n	Mean (SE)	*p*-value	n	Mean (SE)	*p*-value	n	Mean (SE)	*p*-value
**2-min push-ups (numbers)**												
Model 1	765	50.4 (0.41)	<0.01^a^	1009	50.2 (0.36)	0.49^b^	835	48.5 (0.40)	<0.01^c^	818	47.4 (0.40)	<0.01^d^
Model 2	764	50.4 (0.41)	<0.01^a^	1009	50.2 (0.36)	0.79^b^	833	48.5 (0.39)	<0.01^c^	814	47.4 (0.40)	<0.01^d^
Model 3	763	50.4 (0.41)	<0.01^a^	1002	50.1 (0.35)	0.89^b^	826	48.4 (0.39)	0.01^c^	808	47.4 (0.40)	<0.01^d^
Model 4	752	50.4 (0.41)	<0.01^a^	990	50.1 (0.35)	0.95^b^	817	48.5 (0.39)	0.03^c^	797	47.4 (0.40)	<0.01^d^
**2-min sit-ups (numbers)**												
Model 1	765	47.9 (0.28)	<0.01^a^	1009	48.2 (0.25)	0.88^b^	839	47.2 (0.27)	0.03^c^	824	46.9 (0.27)	<0.01^d^
Model 2	764	47.9 (0.28)	<0.01^a^	1009	48.2 (0.25)	0.80^b^	837	47.2 (0.27)	0.05^c^	821	46.9 (0.27)	<0.01^d^
Model 3	763	47.9 (0.28)	0.01^a^	1002	48.1 (0.25)	0.73^b^	829	47.2 (0.27)	0.08^c^	815	46.9 (0.27)	0.01^d^
Model 4	752	47.9 (0.28)	0.11^a^	990	48.2 (0.25)	0.56^b^	820	47.2 (0.27)	0.31^c^	804	46.9 (0.27)	0.11^d^
**3000-m running (seconds)**												
Model 1	708	849.4 (2.55)	<0.01^a^	934	852.6 (2.22)	0.09^b^	743	862.5 (2.49)	<0.01^c^	723	872.6 (2.53)	<0.01^d^
Model 2	707	849.5 (2.50)	<0.01^a^	934	852.6 (2.17)	0.28^b^	741	862.6 (2.44)	<0.01^c^	721	872.8 (2.78)	<0.01^d^
Model 3	706	849.6 (2.50)	<0.01^a^	927	852.8 (2.17)	0.31^b^	734	862.5 (2.44)	<0.01^c^	716	872.8 (2.48)	<0.01^d^
Model 4	697	849.7 (2.48)	<0.01^a^	916	852.8 (2.15)	0.48^b^	725	862.3 (2.41)	<0.01^c^	705	872.5 (2.47)	<0.01^d^

^a^Overall p-value; ^b^WBC: 0^th^-25^th^ % vs. 25^th^-50^th^ %; ^c^WBC: 0^th^-25^th^ % vs. 50^th^-75^th^ %; ^d^WBC: 0^th^-25^th^ % vs. 75^th^-100^th^ %.

Mean ± standard error (SE) for each exercise performance estimated using analysis of covariance with adjustments for Model 1: age and service specialty; Model 2: the covariates in Model 1 and body mass index; Model 3: the covariates in Model 2 and waist circumference; Model 4: the covariates in Model 3, alcohol intake status, cigarette smoking status and weekly physical activity.

### Multiple linear regressions

The results of multiple linear regressions for each exercise performance, with the second lowest, the second highest and the highest normal leukocyte counts in reference to the lowest normal leukocyte counts, are shown in Table 3. The relationships of total leukocyte count and the performance for each exercise in all models are in line with the findings presented in Table 2. In model 4, compared to the lowest normal leukocyte counts, the second highest and highest normal leukocyte counts were inversely correlated with a longer time for a 3000-meter run test (β = 4.93 and 4.65, respectively) and fewer numbers of push-ups in 2 minutes (β = −0.59 and −0.56, respectively). However, there was no relationship between total leukocyte counts and number of sit-ups in 2 minutes in model 4.

**Table 3:** Multiple Liner Regressions of Leukocyte Counts With Each Exercise Performance.

	WBC Counts 25^th^–50^th^ %			WBC Counts 50^th^–75^th^ %			WBC Counts 75^th^–100^th^ %		
	β	95% CI	*p*-value	β	95% CI	*p*-value	β	95% CI	*p*-value
**2-min push-ups (numbers)**									
Model 1	−0.43	−1.49–0.62	0.42	−1.01	−1.57–−0.44	<0.01	−1.03	−1.41–−0.63	<0.01
Model 2	−0.23	−1.28–0.82	0.67	−0.81	−1.37–−0.24	<0.01	−0.77	−1.56–−0.38	<0.01
Model 3	−0.09	−1.23–0.94	0.86	−0.73	−1.28–−0.17	0.01	−0.65	−1.04–−0.26	<0.01
Model 4	−0.03	−1.05–0.99	0.95	−0.59	−1.15–−0.03	0.03	−0.56	−0.96–−0.17	<0.01
**2-min sit-ups (numbers)**									
Model 1	0.06	−0.73–0.85	0.88	−0.44	−0.84–−0.04	0.03	−0.39	−0.65–−0.13	<0.01
Model 2	0.10	−0.69–0.89	0.80	−0.41	−0.81–−0.01	0.04	−0.36	−0.62–−0.95	<0.01
Model 3	0.17	−0.62–0.96	0.67	−0.36	−0.77–0.04	0.07	−0.31	−0.58–−0.05	0.02
Model 4	0.23	−0.55–1.01	0.56	−0.20	−0.62–0.20	0.31	−0.21	−0.48–0.05	0.11
**3000-m run (seconds)**									
Model 1	5.46	−1.08–12.01	0.10	7.39	3.98–10.78	<0.01	8.10	5.62–10.59	<0.01
Model 2	3.45	−2.97–9.87	0.29	5.94	2.56–9.32	<0.01	6.03	3.58–8.49	<0.01
Model 3	3.19	−3.21–9.58	0.32	5.72	2.36–9.08	<0.01	5.79	3.33–8.24	<0.01
Model 4	2.58	−3.67–8.84	0.41	4.93	1.61–8.25	<0.01	4.65	2.20–7.10	<0.01

Data are presented as β-value and 95% confidence intervals (CI) using Pearson’s correlation coefficient for Model 1: age and service specialty; Model 2: the covariates in Model 1 and body mass index; Model 3: the covariates in Model 2 and waist circumference; Model 4: the covariates in Model 3, alcohol intake status, cigarette smoking status and weekly physical activity.

### Subgroup analysis based on age

Table 4 reveals the results of multiple linear regressions for total leukocyte counts and exercise performance in males aged ≥30 years and in males aged <30 years for each exercise. The results were similar in the inverse association between the highest normal leukocyte counts and exercise performance for those aged ≥30 years and those aged <30 years in model 4. However, the inverse relationship of the second highest normal leukocyte counts with exercise performance was not significant in those aged <30 years in model 4 for any exercise.

**Table 4:** Multiple Liner Regressions of Leukocyte Counts With Each Exercise Performance Stratified by Age.

	N	WBC Counts 25^th^–50^th^ %			WBC Counts 50^th^–75^th^ %			WBC Counts 75^th^–100^th^ %		
		β-value	95% CI	*p*-value	β-value	95% CI	*p*-value	β-value	95% CI	*p*-value
**2-min push-ups (numbers)**										
30–50 y/o	1787	−0.43	−1.66–0.80	0.49	−0.94	−1.58–−0.30	<0.01	−0.51	−0.94–−0.07	0.02
18–29 y/o	1640	0.59	−1.07–2.24	0.48	−0.06	−0.97–0.86	0.90	−0.58	−1.25–0.09	0.09
**2-min sit-ups (numbers)**										
30–50 y/o	1794	0.08	−0.96–1.10	0.88	−0.50	−1.02–0.02	0.06	−0.24	−0.59–0.11	0.18
18–29 y/o	1643	0.50	−0.67–1.68	0.40	0.16	−0.45–0.76	0.61	−0.19	−0.59–2.14	0.35
**3000-m run (seconds)**										
30–50 y/o	1598	4.42	−3.75–12.58	0.28	5.40	1.29–9.51	0.01	4.93	1.75–8.09	<0.01
18–29 y/o	1510	−0.36	−9.97–9.26	0.94	4.10	−1.19–9.40	0.12	4.14	0.32–7.95	<0.001

Data are presented as β-value and 95% confidence intervals (CI) using Pearson’s correlation coefficient for Model 4: age, service specialty, body mass index, waist circumference, alcohol intake status, cigarette smoking status and weekly physical activity.

## Discussion

Our principal findings were that systemic low-grade inflammation as reflected by total leukocyte counts within normal limits was inversely associated with aerobic and anaerobic fitness in healthy young male military members in Taiwan. Higher total leukocyte counts were correlated with longer times to complete a 3000-meter run, as an evaluation of aerobic fitness, and fewer push-ups in 2 minutes, as an evaluation of anaerobic fitness.

Notably, both maximal aerobic and anaerobic fitness were decreased in male military members with higher normal leukocyte counts (6,500–9,999/mm^3^) compared to those with lower normal leukocyte counts (4,000–6,499/mm^3^), indicating that a total leukocyte count of 6,500/mm^3^ could be thought of as the level at which low-grade inflammation impairs both muscular strength and cardiorespiratory fitness. Although the types, amount and frequency of daily physical activity were similar among military members, an inverse relationship between total leukocyte counts within normal limits and the performance of 3000-m run and 2-minute push-up tests remained remarkable. In addition, the inverse relationship was present independent of body adiposity, habitual tobacco smoking and alcohol intake, which are the most common causes of low-grade inflammation and impaired physical fitness among young adults free of chronic diseases^7,8^.

Numerous studies have revealed an association of low-grade inflammation with low physical activity, greater body fat, insulin resistance and a higher risk of type 2 diabetes and cardiovascular disease^28–32^. These might be the mechanisms for the inverse relationship between total leukocyte counts and cardiorespiratory fitness in middle- and old-aged individuals in previous population studies^13–16^. However, most of the studies did not control for baseline confounders, such as physical activity and habitual toxic agent use (e.g., smoking and alcohol) status. Although some reports also revealed that levels of physical fitness were associated with C-reactive protein and C3 complement concentrations among young adults or prepubertal children^28,33^, most of the studies were limited by a small sample size and by the method of model adjustment only controlling for body adiposity and not for other confounding factors. The findings of this study were consistent with previous study results that total leukocyte counts, which are highly related to C-reactive protein and C3 complement concentrations, could also be used for evaluating the superiority of physical fitness in young adults.

With regard to the relationship between low-grade inflammation status and anaerobic fitness (muscular strength), the inverse association was present in both the 2-minute push-up and 2-minute sit-up tests and was independent of the demographic and anthropometric indexes. However, the relationship became nonsignificant for the 2-minute sit-up test with additional adjustment for physical activity and toxic agent use. The association differed for the two anerobic exercise tests, possibly due to a variation in intensity of training and the amount of current tobacco smoking and alcohol intake. In addition, we found that the threshold for a significant inverse association between total leukocyte count and physical fitness was increased to 7.5 × 10^3^/mm^3^ in the highest category in individuals aged 18–29 years. This finding could be the reason that a mild elevation in total leukocyte count at baseline was present in the younger participants; the elevation might be caused by a greater immune response to environmental stimuli and physical training rather than low-grade inflammation in the body, and thus, the association of the second highest total leucocyte count with physical fitness was null in those males aged less than 30 years in the present study.

Our study had several strengths. First, both routine blood examinations and exercise tests were performed strictly, and all of the procedures were performed in a standard manner. Second, since there were numerous male military members included in the study, the power was sufficient to detect differences among groups. Third, many unmeasured confounders in this study would be minimized as daily schedules in the military are similar. In contrast, our study had some limitations. First, the study included only young physically fit male subjects, and it is difficult to apply the results to young female subjects, older individuals, and those with overall average or below-average fitness. Second, since the participation rate of male military members in the CHIEF study was 66.5%, we could not exclude the possibility of selection bias. Third, although many covariates were controlled at baseline, the presence of potential confounders such as social/mental stress, medication use, and musculoskeletal conditions, which might result in a bias, could not be avoided. Fourth, we did not have information regarding the subtypes of leucocytes, and the total leukocyte count might vary with different situations, such as changes in weather, despite blood samples being drawn in the same place with good air conditioning in accordance with the rules for laboratory examinations. Finally, the statistical analyses failed to correct for multiple comparisons.

In conclusion, our findings suggested that there was an inverse association of total leukocyte counts within normal limits and aerobic fitness as well as parts of anaerobic fitness in young male military members in Taiwan who underwent rigorous training regularly. In addition, the temporal association between total leucocyte count and physical fitness needs confirmation in longitudinal studies.
